# The causal relationship between neuromyelitis optica spectrum disorder and other autoimmune diseases

**DOI:** 10.3389/fimmu.2022.959469

**Published:** 2022-09-29

**Authors:** Xiaofei Wang, Ziyan Shi, Zhengyang Zhao, Hongxi Chen, Yanlin Lang, Lingyao Kong, Xue Lin, Qin Du, Jiancheng Wang, Hongyu Zhou

**Affiliations:** ^1^ Department of Neurology, West China Hospital, Sichuan University, Chengdu, China; ^2^ Mental Health Centre and Psychiatric Laboratory, West China Hospital, Sichuan University, Chengdu, China

**Keywords:** autoimmune diseases, Mendelian randomization, neuromyelitis optica spectrum disorders, genome-wide associated study, causal relation

## Abstract

**Objectives:**

The coexistence of neuromyelitis optica spectrum disorder (NMOSD) with other autoimmune diseases has been well recognized. However, the causal association between these two conditions has not been fully studied. The etiology and therapies of NMOSD coexisting with autoimmune diseases also need to be elucidated.

**Methods:**

We performed two-sample Mendelian randomization (MR) analysis to examine the causality. Genome-wide association (GWAS) summary data from NMOSD, autoimmune thyroid disease (AITD), systemic lupus erythematosus (SLE), and Sjogren’s syndrome (SS) were used to identify genetic instruments. Causal single-nucleotide polymorphisms (SNPs) were annotated and searched for cis-expression quantitative trait loci (cis-eQTL) data. Pathway enrichment analysis was performed to identify the mechanism of NMOSD coexisting with AITD, SLE, and SS. Potential therapeutic chemicals were searched using the Comparative Toxicogenomics Database.

**Results:**

The MR analysis found that AITD, SLE, and SS were causally associated with NMOSD susceptibility, but not vice versa. Gene Ontology (GO) enrichment analysis revealed that MHC class I-related biological processes and the interferon-gamma-mediated signaling pathway may be involved in the pathogenesis of NMOSD coexisting with AITD, SLE, and SS. A total of 30 chemicals were found which could inhibit the biological function of cis-eQTL genes.

**Conclusions:**

Our findings could help better understand the etiology of NMOSD and provide potential therapeutic targets for patients with coexisting conditions.

## Introduction

Neuromyelitis optica spectrum disorder (NMOSD) is an autoimmune demyelinating disease of the central nervous system (CNS), which may lead to blindness and paralysis (1). Epidemiological studies show that some peripheral autoimmune diseases (ADs) may coexist with NMOSD, including systemic lupus erythematosus (SLE), Sjogren’s syndrome (SS), and autoimmune thyroid disease (AITD) (2, 3). This phenomenon makes us curious whether there is a shared pathology between NMOSD and other ADs. There is a certain significance in answering this question for understanding the pathogenesis of autoimmune diseases.

Recently, the Mendelian randomization (MR) method has been widely used to investigate the causal relationships between two diseases using summary statistic data from genome-wide association studies (GWAS). Few studies apply this method to the field of NMOSD. Since the coexistence of NMOSD and ADs can be seen clinically, herein we use MR analysis to investigate the genetic relationships between those two conditions. We found that the activation of MHC class I-related biological processes and the interferon-mediated signaling pathway may be the shared pathology of NMOSD coexisting with other ADs. We also predicted 30 potential therapeutic chemicals for the coexisting condition. Our study provides new insights to the pathogenesis and treatment of NMOSD.

## Methods

### GWAS summary statistic data

**Figure 1:**
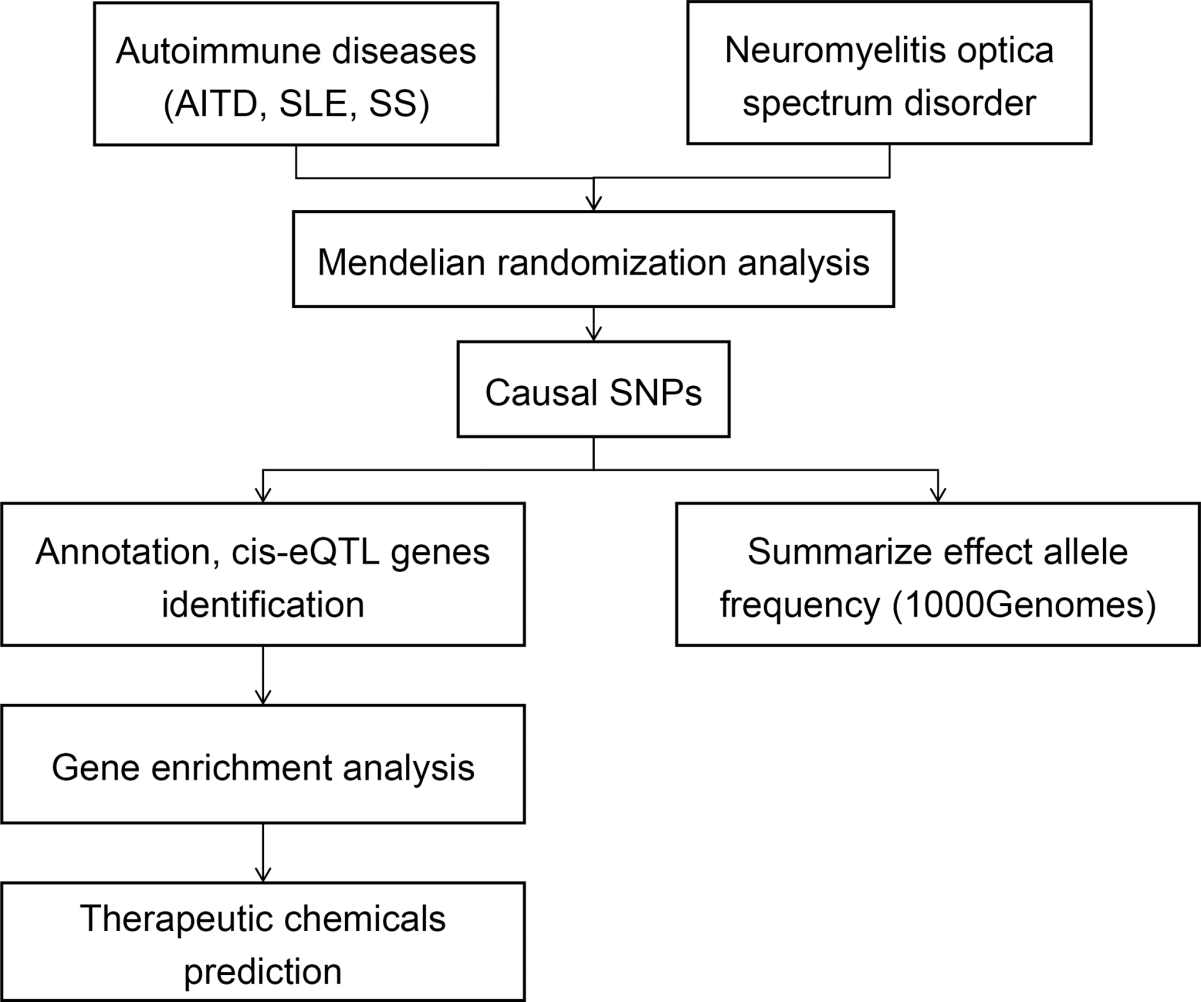
Flowchart of the study’s design. Summary data of GWAS traits were extracted, then MR analysis was performed to detect the causal relationships between NMOSD and other ADs (AITD, SLE, and SS). The frequencies of causal SNPs were searched in 1000 Genomes. The causal SNPs were annotated, and gene enrichment analysis was performed. Chemicals that may affect the enriched pathways were analyzed.

**Figure 1** shows the overall design of this study. We investigated the genetic causality between NMOSD and three other ADs that most commonly coexist with NMOSD, including AITD, SLE, and SS. All GWAS summary statistic data were downloaded from the GWAS catalog (4). The most recent traits with European ancestry and the largest sample size were used. The NMOSD trait (GCST005964) includes 215 patients and 1,244 controls. The AITD trait (GCST90014440) includes 859 patients and 324,074 controls. The SLE trait (GCST90014462) includes 624 patients and 324,074 controls. The SS trait (GCST90014460) includes 647 patients and 324,074 controls.

### Genetic instrument selection

The single-nucleotide polymorphism (SNP) used as the exposure instrumental variables (IVs) of AD were selected with a p value less than 5E-8. Considering the lower sample size of the NMOSD trait, the p value of exposure IVs was set as 1E-6. Then, the linkage disequilibrium in the selected IVs was clumped with r^2^ <0.1 based on the 1000 Genomes Project. The F-statistics of each selected IVs were calculated using mRnd (5) and would be excluded if F-statistics <10.

### Two-sample MR analysis

Two-sample MR analyses were performed using five models (MR–Egger, weighted median, inverse variance weighted (IVW), simple mode, and weighted mode) (6, 7). The palindromic SNPs with intermediate allele frequencies were excluded from MR analysis. We performed Cochran’s Q test to evaluate heterogeneity and used the p value for the intercept in MR–Egger regression to assess the pleiotropic effect. All analyses were performed using the TwoSampleMR package (version 0.5.6) of R software (version 4.1.2).

### Functional evaluation of causal SNPs

We used the ‘mr_singlesnp’ function in the TwoSampleMR package to extract the basic information of causal SNPs (rsID, location, effect allele). Then, the closest genes of the extracted SNPs were annotated in the dbSNP database (8) and GWAS 4D (9). The frequencies of causal SNPs in different populations were recorded with the reference of the 1000 Genomes Project. To investigate whether causal SNPs affect gene expression, we analyzed cis-eQTL in whole blood from the GTEx Portal (9). cis-eQTL genes with p value <0.05/gene number and normalized effect size (NES) >0 were identified as significant genes and enrolled in enrichment analysis. We used DAVID 6.8 (10) to perform Gene Ontology (GO) enrichment analysis and Kyoto Encyclopedia of Genes and Genomes (KEGG) pathway enrichment analysis.

### Potential therapeutic chemical prediction

To predict potential therapeutic chemicals for NMOSD coexisting with AD, we assessed the Comparative Toxicogenomics Database (CTD) (11). We used GO:id as the query condition and selected chemicals that have clear evidence to inhibit the GO function of cis-eQTL genes. Then, the chemical list was manually reviewed with reference of the PubChem database (12), and information such as pharmacology, biochemistry properties, FDA UNII, and current clinical applications was recorded. Chemicals were excluded if evidence shows that they may promote the expression of IL-6 or IL-17 (key cytokines in the pathogenic process of NMOSD). Cytoscape software (version 3.9.1) was used to construct the chemical–gene interaction network.

## Results

### The presence of ADs may facilitate NMOSD pathology

**Figure 2:**
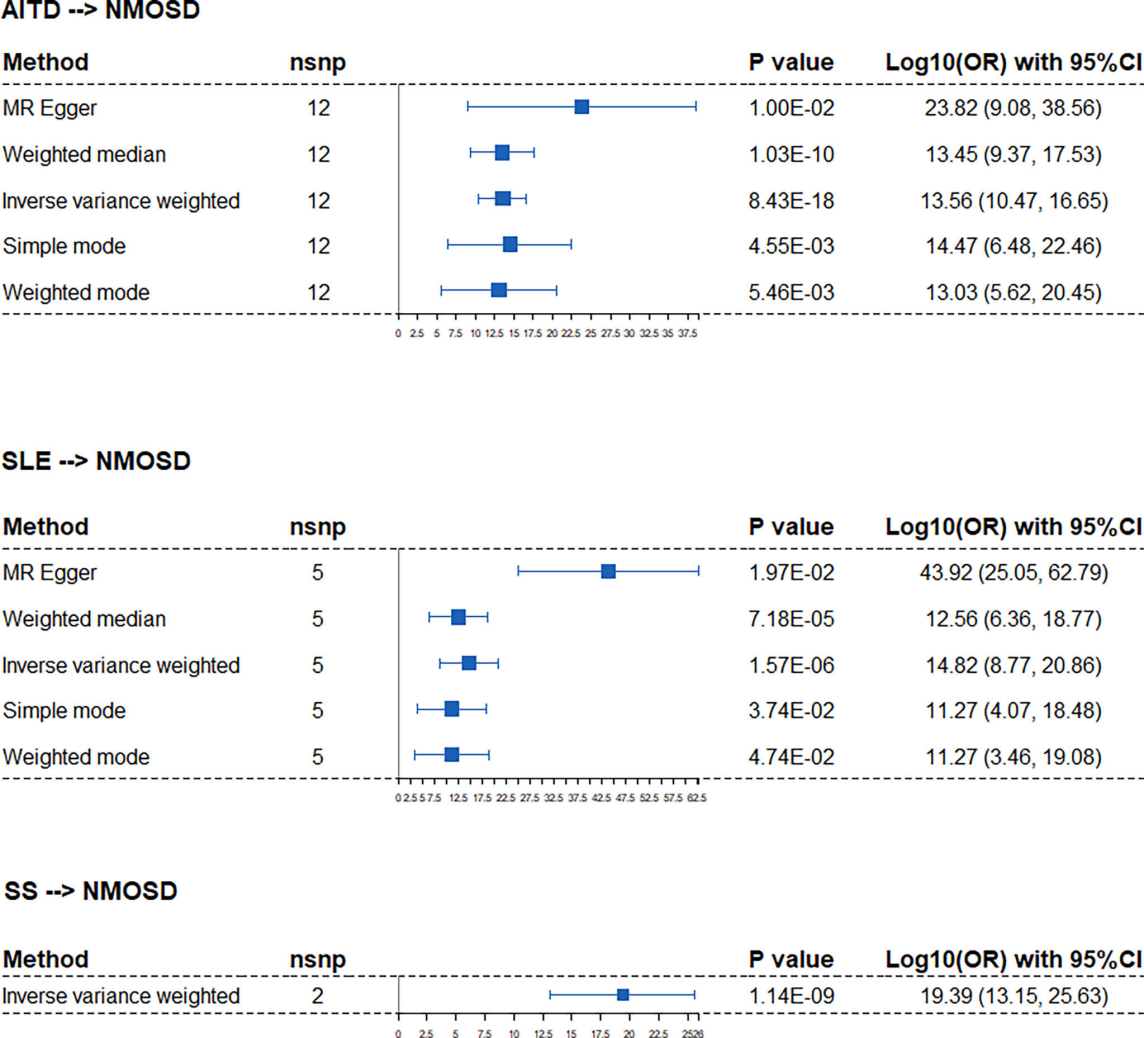
Forest plots of Mendelian randomization analysis that estimates the risk of NMOSD on the exposure of AITD, SLE, and SS. nsnp, number of causal SNPs; CI, confidence interval.

We identified significant causality between NMOSD and other ADs, but the causal effects were stronger on the risk of NMOSD. The IVW analysis showed that the risk of NMOSD was increased on the exposure of AITD odds ratio [(OR): 3.62E13, 95% confidence interval (CI): 2.92E10 to 4.48E16], SLE (OR: 66.55E14, 95% CI: 5.87E08 to 7.31E20), and SS (OR: 2.46E19, 95% CI: 1.41E13, 4.29E25) (**Figure 2**). Among the above three exposure conditions, SS did not have enough IVs to perform analysis other than IVW. Except for SS, the MR–Egger regression analysis, weighted median estimator, simple mode, and weighted mode of AITD and SLE all provide results of the same direction and magnitude as IVW analysis, which indicates a stable and strong causality in the risk of developing NMOSD. The heterogeneity and pleiotropy were not found (**Table 1**).

**Table 1:** Heterogeneity and pleiotropy tests for the associations of autoimmune diseases with NMOSD.

Exposure	Q-value	P_Q_	Intercept	P_intercept_
AITD	14.014	0.172	-0.275	0.194
SLE	0.686	0.876	-0.804	0.054
SS	0.627	0.429	–	–

AITD, autoimmune thyroid disease; SLE, systemic lupus erythematosus; SS, Sjogren syndrome; NMOSD, neuromyelitis optica spectrum disorder.

**Figure 3:**
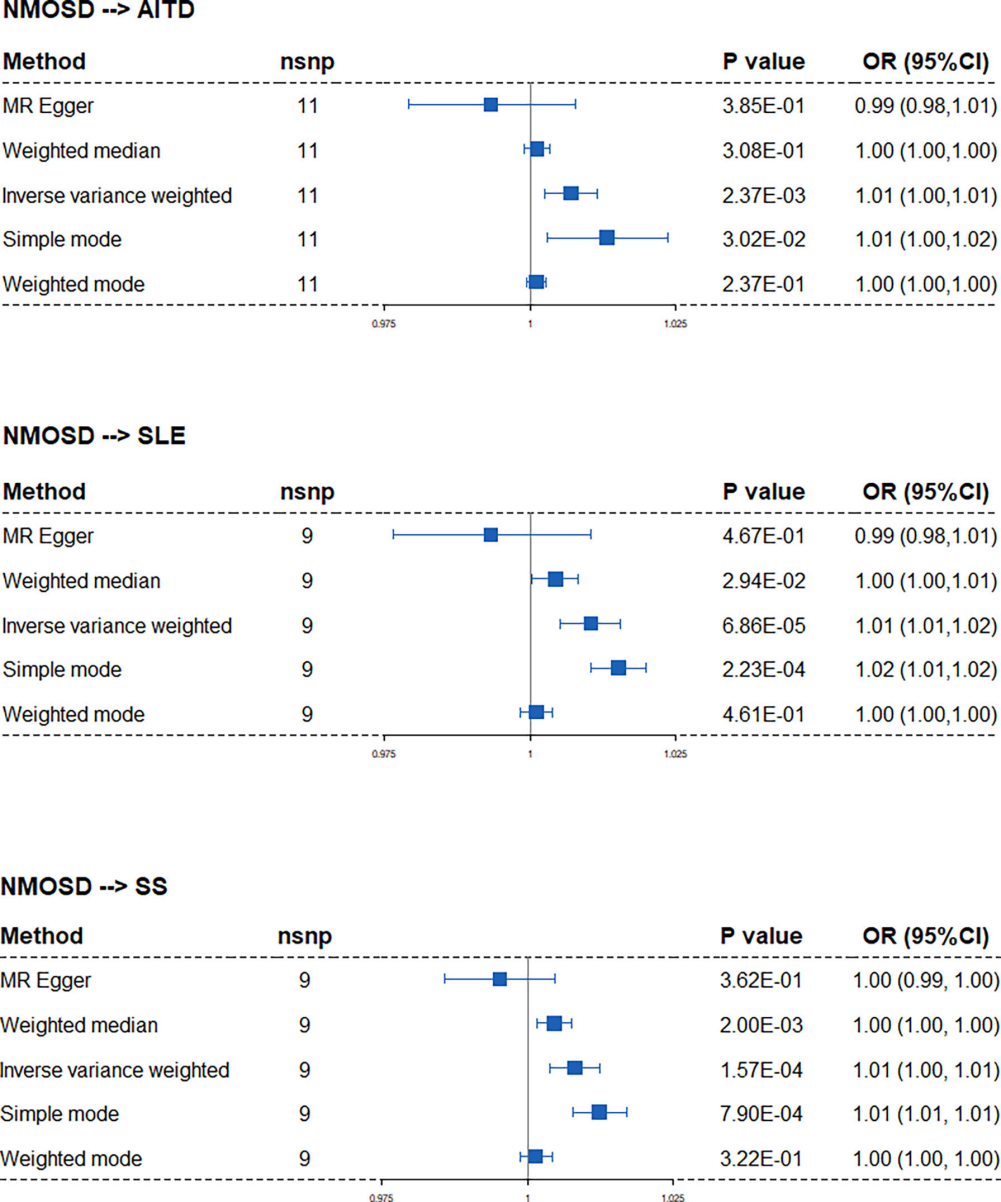
Forest plots of Mendelian randomization analysis that estimates the risk of AITD, SLE, and SS on the exposure of NMOSD. nsnp, number of causal SNPs; CI, confidence interval.

When applying NMOSD as the exposure condition, the IVW analysis showed increased risks of developing AITD (OR: 1.01, 95% CI: 1.00 to 1.01), SLE (OR: 1.01, 95% CI: 1.01 to 1.02), and SS (OR: 1.01, 95% CI: 1.00 to 1.01) (**Figure 3** **)**. However, neither the weighted median estimator nor the weighted mode reached a statistical significance with p value >0.05/5 (Bonferroni correction). Besides, the MR–Egger regression analysis showed contrasting trends. Taken together, the presence of AITD, SLE, and SS may facilitate NMOSD pathology but not vice versa.

### Higher frequencies of causal SNPs in East Asia were found

The below sections focused on the exposure of AITD, SLE, and SS on NMOSD. Obvious differences in the frequencies of causal SNPs between East Asian and Caucasian were found (**Table 2**), which may explain why the incidence of NMOSD is higher in the East Asia region. However, minimal difference in the frequencies of causal SNPs was noticed between genders (**Table 3**).

**Table 2:** Frequencies of causal SNPs of NMOSD on the exposure of autoimmune diseases based on regions.

SNP	Effect allele	Effect allele frequency			
		Global	Europe	America	East Asia
***AITD ➔ NMOSD***					
rs17206350	C	0.581	0.497	0.503	0.677
rs2239701	C	0.487	0.451	0.513	0.537
rs2248372	A	0.315	0.398	0.385	0.215
rs241438	T	0.375	0.359	0.382	0.517
rs2523545	A	0.759	0.651	0.758	0.898
rs2736426	T	0.635	0.551	0.605	0.584
rs2894053	A	0.190	0.283	0.308	0.043
rs3095303	A	0.287	0.324	0.310	0.095
rs3132948	G	0.578	0.464	0.572	0.635
rs429150	C	0.525	0.511	0.481	0.547
rs805262	T	0.382	0.473	0.416	0.592
rs9268235	T	0.020	0.079	0.019	0.001
***SLE ➔ NMOSD***					
rs1610615	T	0.360	0.285	0.370	0.408
rs2517485	T	0.362	0.346	0.427	0.186
rs497309	C	0.031	0.074	0.023	0
rs6904670	G	0.421	0.446	0.409	0.328
rs805262	T	0.382	0.473	0.416	0.592
***SS ➔ NMOSD***					
rs1264347	T	0.042	0.081	0.023	0.024
rs429150	C	0.525	0.511	0.481	0.547

AITD, autoimmune thyroid disease; SLE, systemic lupus erythematosus; SS, Sjogren syndrome; SNP, single-nucleotide polymorphism; NMOSD, neuromyelitis optica spectrum disorder.

**Table 3:** Frequencies of causal SNPs of NMOSD on the exposure of autoimmune diseases based on genders.

SNP	Effect allele	Effect allele frequency							
		Global		Europe		America		East Asia	
		Male	Female	Male	Female	Male	Female	Male	Female
***AITD ➔ NMOSD***									
rs17206350	C	0.587	0.575	0.513	0.483	0.509	0.497	0.674	0.679
rs2239701	C	0.477	0.496	0.442	0.46	0.471	0.554	0.533	0.54
rs2248372	A	0.319	0.314	0.408	0.388	0.374	0.395	0.189	0.24
rs241438	T	0.369	0.38	0.346	0.371	0.391	0.373	0.496	0.537
rs2523545	A	0.763	0.755	0.629	0.671	0.774	0.743	0.893	0.902
rs2736426	T	0.649	0.622	0.563	0.54	0.609	0.602	0.594	0.575
rs2894053	A	0.194	0.186	0.285	0.281	0.338	0.28	0.049	0.037
rs3095303	A	0.285	0.289	0.31	0.337	0.297	0.322	0.096	0.094
rs3132948	G	0.575	0.582	0.477	0.452	0.574	0.571	0.654	0.617
rs429150	C	0.536	0.514	0.519	0.504	0.479	0.483	0.561	0.533
rs805262	T	0.384	0.38	0.485	0.462	0.424	0.41	0.555	0.627
rs9268235	T	0.019	0.02	0.073	0.084	0.021	0.017	0	0.002
***SLE ➔ NMOSD***									
rs1610615	T	0.367	0.352	0.281	0.289	0.371	0.37	0.439	0.379
rs2517485	T	0.365	0.359	0.331	0.359	0.418	0.435	0.191	0.181
rs497309	C	0.033	0.029	0.071	0.076	0.026	0.02	0	0
rs6904670	G	0.425	0.418	0.46	0.433	0.397	0.421	0.34	0.317
rs805262	T	0.384	0.38	0.485	0.462	0.424	0.41	0.555	0.627
***SS ➔ NMOSD***									
rs1264347	T	0.041	0.042	0.081	0.08	0.029	0.017	0.025	0.023
rs429150	C	0.536	0.514	0.519	0.504	0.479	0.483	0.561	0.533

AITD, autoimmune thyroid disease; SLE, systemic lupus erythematosus; SS, Sjogren syndrome; SNP, single-nucleotide polymorphism; NMOSD, neuromyelitis optica spectrum disorder.

### NMOSD coexisting with ADs may be mediated by the antigen processing, interferon, and complement system

**Figure 4:**
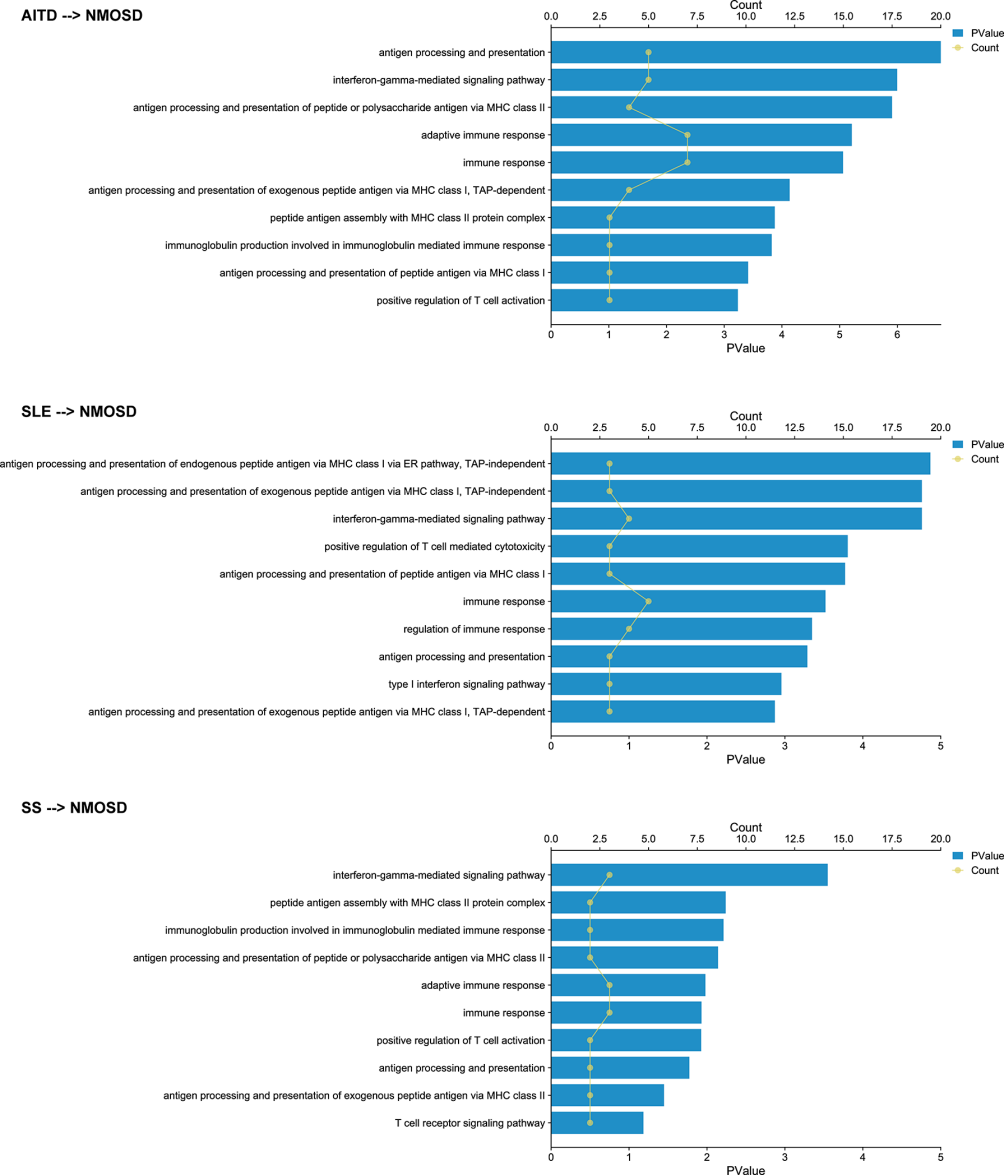
Gene Ontology enrichment analysis of the cis-eQTL genes. Dots indicate the number of genes that enriched in certain pathways; columns indicate the - log10(p-value) of pathway enrichment analysis.

When annotating the causal SNPs using the dbSNP database and GWAD 4D (**Table 4** and **Additional File 1**), genes related to class I major histocompatibility complex (MHC) (TAP2, HCP5, BAG6), MHC-II (HLA-DQB3), and the complement system (C2, CFB) were found to be associated with the pathology of NMOSD that coexists with other ADs. To further clarify the function of the causal SNPs, we first searched cis-eQTL in whole blood (**Table 4** and **Additional File 1**). Then, we performed pathway enrichment using significant cis-eQTL genes with NES >0 (**Figure 4**). GO enrichment analyses showed that MHC class I-related biological processes, type I interferon signaling pathway, and complement activation were upregulated when NMOSD was followed by the presence of AITD or SLE. On the contrary, MHC class II-related biological processes and the interferon-gamma-mediated signaling pathway are in the leading roles in NMOSD coexisting with SS. KEGG enrichment analyses identified two types of pathway: autoimmune disease-related pathway and virus infection-related pathway (**Additional File 2**).

**Table 4:** eQTLs of causal SNPs of NMOSD on the exposure of autoimmune diseases.

SNP	Closest gene	cis-eQTL gene (NES >0)
***AITD ➔ NMOSD***		
rs17206350	MTCO3P1; HLA-DQB3	AIF1; ATP6V1G2; BAG6 C2; C4B; CCHCR1; CYP21A1P CYP21A2; FLOT1 HCG27; HCG27; HLA-B; HLA-C HLA-DQA2; HLA-DQA2 HLA-DQB2; HLA-DQB2 HLA-DRB1; HLA-DRB6 HLA-DRB9; HLA-S IER3; LINC00243; LY6G5B LY6G5C; MICB; MIR6891 NOTCH4; POU5F1; PRRC2A PSMB9; PSMB9; PSORS1C3 STK19B; TAP2 XXbac-BPG248L24.12; ZBTB12
rs2239701	TAP2	
rs2248372	HCP5	
rs241438	TAP2	
rs2523545	DHFRP2; RNU6-283P	
rs2736426	VWA7; VARS1	
rs2894053	–	
rs3095303	–	
rs3132948	–	
rs429150	TNXB	
rs805262	BAG6; C6orf47; GPANK1	
rs9268235	TSBP1; TSBP1-AS1	
***SLE ➔ NMOSD***		
rs1610615	HLA-F-AS1	AIF1; BAG6; C2; C4B; CCHCR1 CYP21A2; GABBR1; HCG4P3 HCP5B; HLA-C; HLA-DRB1 HLA-F; HLA-H; HLA-U LY6G5B; LY6G5C; MICB POU5F1; PRRC2A; PSORS1C3 RPL23AP1; XXbac-BPG248L24.12 ZFP57
rs2517485	C6orf15	
rs497309	C2	
rs6904670	CFB	
rs805262	BAG6; C6orf47; GPANK1	
***SS ➔ NMOSD***		
rs1264347	LINC00243	ATP6V1G2; CCHCR1; CYP21A1P FLOT1; HCP5B; HLA-C; HLA-DQA2 HLA-DQB2; HLA-DRB6; HLA-S IER3; LINC00243; LY6G5B PRRC2A; XXbac-BPG248L24.12
rs429150	TNXB	

AITD, autoimmune thyroid disease; SLE, systemic lupus erythematosus; SS, Sjogren syndrome; NMOSD, neuromyelitis optica spectrum disorder.

### Potential therapeutic chemicals for NMOSD coexisting with other AD

**Figure 5:**
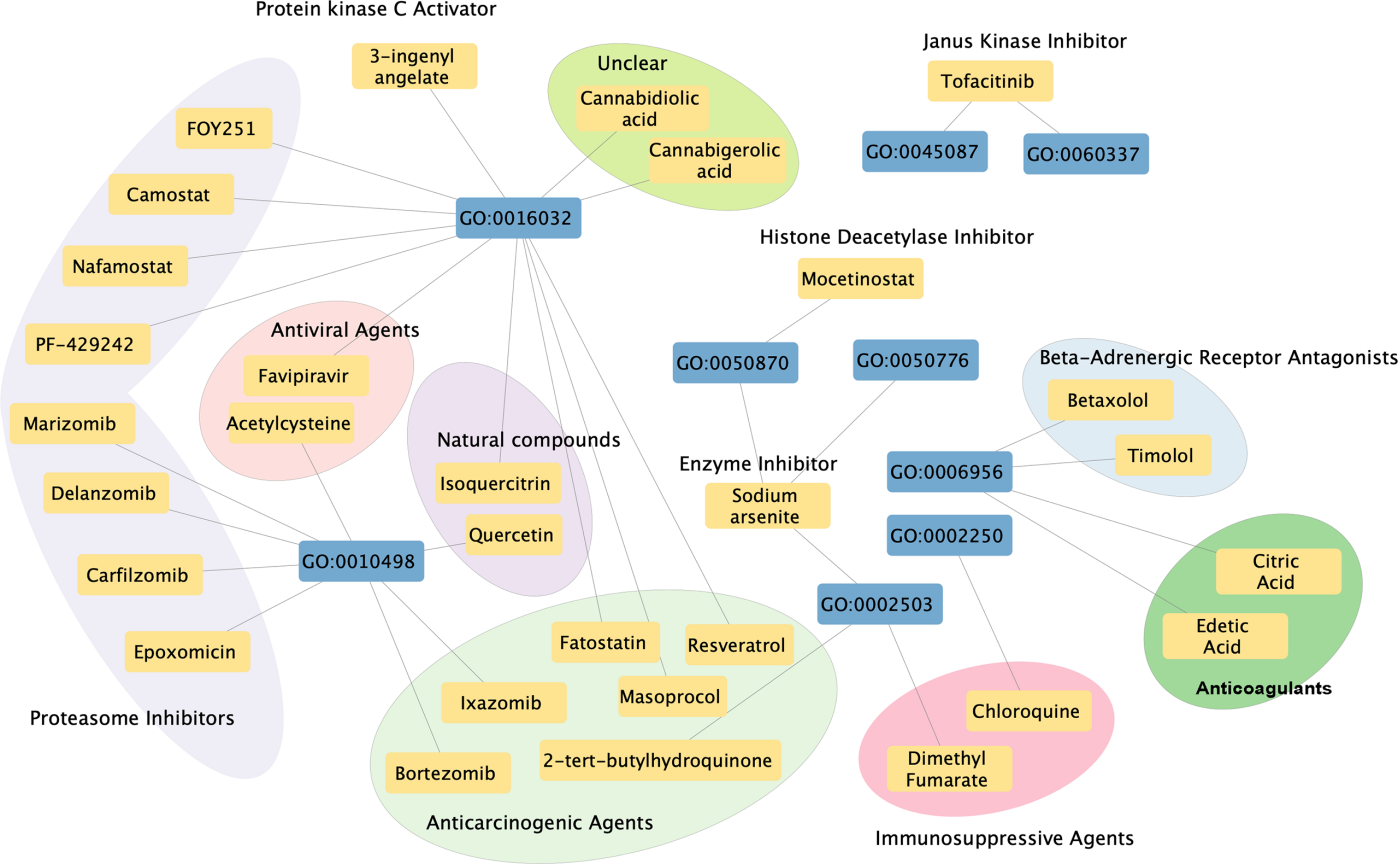
Chemical–biological process interaction network. Blue boxes indicate significant enriched GO pathways, and yellow boxes indicate potential therapeutic chemicals. Each line indicates an interaction between the GO pathway and chemical. Circles indicate chemicals that serve the similar function.

A total of 30 chemicals were found which could inhibit the biological function of cis-eQTL genes (**Figure 5**). These chemicals include proteasome inhibitors (e.g., carfilzomib, which has been used in treating lymphoma and leukemia), anticarcinogenic agents, traditional immunosuppressive agents [e.g., dimethyl fumarate (DMF), which has been used in treating multiple sclerosis], antiviral agents, Janus kinase (JAK) inhibitor (tofacitinib, has been used in treating SLE, SS, and other connective tissue diseases), natural compounds, anticoagulants, beta-adrenergic receptor antagonist, enzyme inhibitor, histone deacetylase inhibitor, and protein kinase c activator. A detailed list of the information and clinical usage of these chemicals can be found in **Additional File 3**.

## Discussion

The coexistence of NMOSD with other autoimmune diseases has been well recognized. The literature review performed by Sareh et al. (2) shows that SLE, SS, and AITD are the most common disorders associated with NMOSD. The International Panel for NMOSD Diagnosis (IPND) suggested in 2015 that the SLE or SS would be a coexistence rather than a complication, and the presence of these ADs strengthens the confidence of NMOSD diagnosis (13). The coexistence of NMOSD and ADs indicates a shared pathology between these two conditions, possibly due to genetic tendencies toward an abnormal immune system. However, there is no evidence regarding which condition occurs first. While several studies reported that NMOSD occurred following the presence of ADs (14, 15) and even AQP4-IgG is present in the serum years before the first NMODS attack (16, 17), there is also evidence suggesting that ADs are a consequence of NMOSD (18–21). Our current MR study, for the first time, provides evidence that AITD, SLE, and SS may facilitate NMOSD pathology but not vice versa.

**Figure 6:**
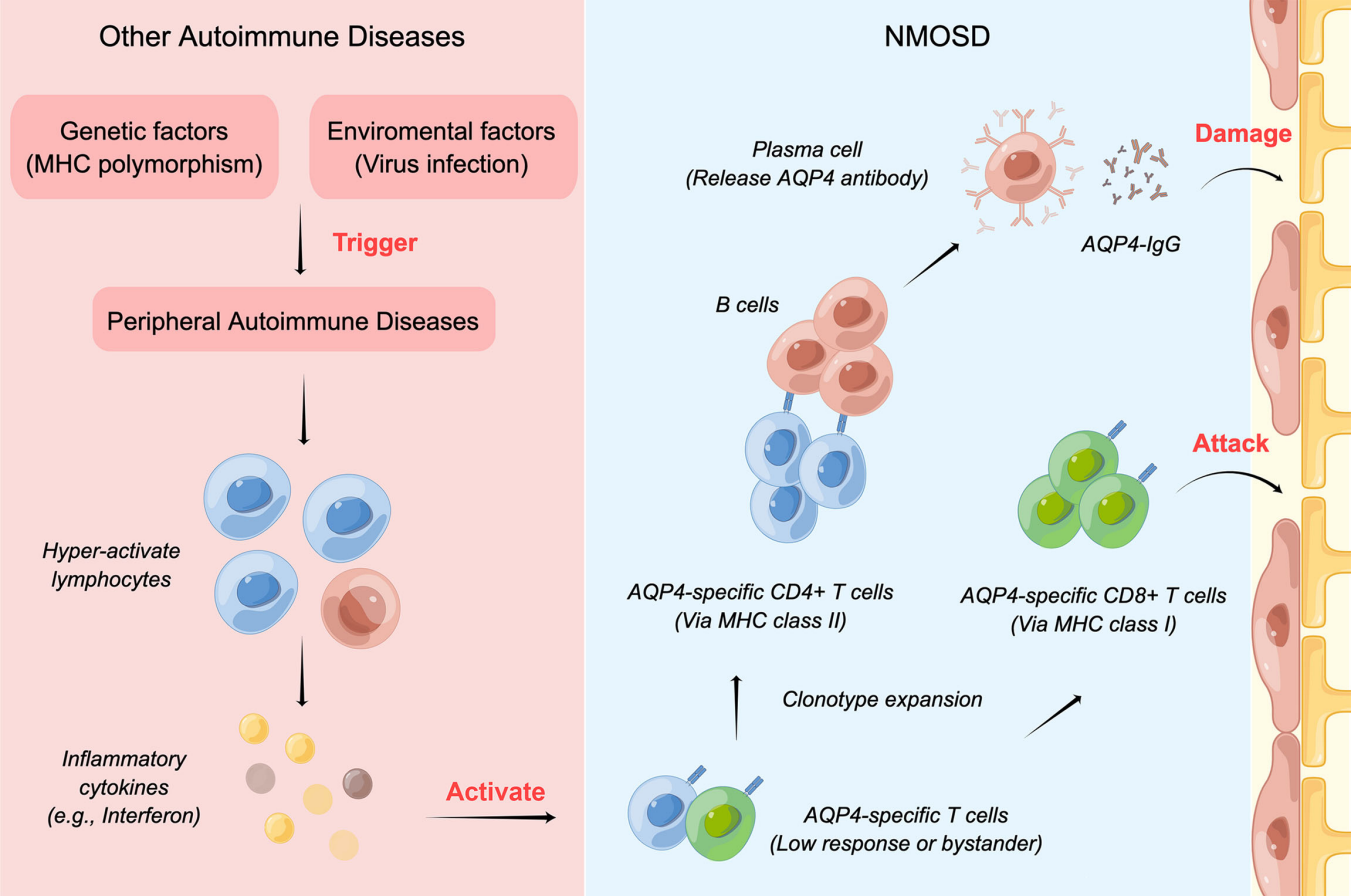
The mechanism of NMOSD coexisting with other ADs. Both environmental and genetic factors contribute to the pathogenesis of peripheral ADs. After peripheral ADs occur, lymphocytes may become hyperactive and produce high levels of inflammatory cytokines, which would activate the low-response or bystander T cells specific to AQP4. Then, the astrocytes would be attacked by AQP4-specific CD8+ T cells and the AQP4-IgG antibody.

Both environmental (e.g., viral infection) and genetic factors (e.g., polymorphism of MHC region) contribute to the pathogenesis of peripheral ADs. However, it is unclear how the peripheral ADs may facilitate the onset of NMOSD. After peripheral ADs occur, the B and T lymphocytes may become hyperactive and produce high levels of inflammatory cytokines. In the next step, the low-response or bystander T cells specific to AQP4, which has been in the body since maturation, may be activated, and astrocytes may be attacked by AQP4-specific CD8^+^ T cells and the AQP4-IgG antibody (22). **(** **Figure 6** **).** Our study found that IFN signaling pathways were upregulated under the comorbidity condition, which may facilitate the activation of T cells and induce the expression of the MHC–peptide complex on normal cells (22), again aggravating astrocyte damage and demyelination.

It is well recognized that MHC class II-related biological processes have leading roles in the etiopathogenesis of NMOSD. The T-cell receptor (TCR) on CD4^+^ T cells combined with peptides forms a complex with MHC class II molecules and then provides a second signal for B-cell activation. Interestingly, the current study identified that MHC class I-related biological processes may be involved in the pathogenesis of NMOSD coexisting with other ADs, which suggests an enhanced function of CD8^+^ T cells in those patients. Recent studies of our team have proven that CD8^+^ T cells participate in the pathology of NMOSD as well (23, 24).

Endogenous antigens (usually produced after virus infection) are presented to CD8^+^ T cells in the form of the peptide–MHC class I molecule complex. The Epstein–Barr virus (EBV) has been recognized as a driver of multiple sclerosis (25, 26), while its role in NMOSD remains unclear. Saeko et al. (27) showed that the CSF anti-EA-IgG titers were also higher in patients with NMOSD than in those with multiple sclerosis. Justin et al. (28) reported a case of NMOSD coexisting with SS whose CSF was positive for EBV. In our study, pathways related to virus infection, such as EBV infection, were also enriched. The above evidence suggests that persistent EBV replication may contribute to the pathogenesis of NMOSD coexisting with ADs.

As for the treatments, whether patients with coexisting conditions should receive the NMOSD recipe or the AD recipe or a different recipe remains unknown. One thing is for sure that tumor necrosis factor-a (TNF-a) monoclonal antibodies (mAbs) should be avoided because they increase the risk of demyelinating events (29–32). Other than that, rituximab (33) (34), cyclophosphamide (35), and tocilizumab (36) have shown satisfied results. Our current study predicted 30 potential therapeutic chemicals based on the biological processes of cis-eQTL genes, including DMF. DMF is one of the disease-modifying therapies (DMTs) for multiple sclerosis. It is well recognized that DMT will exacerbate the disease severity of NMOSD, but there is a lack of evidence regarding the effects of DMF in treating NMOSD (37). DMF mainly suppresses the function of interferon gamma and reduces the proportion of circulating Th1 (38, 39), and our current study revealed that the interferon-gamma-mediated pathway was involved in NMOSD coexisting with ADs. Therefore, DMF might be a potential therapeutic chemical for NMOSD coexisting with ADs, but the efficacy needs to be verified in *in vivo* and *ex vivo* studies.

Based on the GWAS summary data, our study for the first time demonstrated strong causal effects of AITD, SLE, and SS on the risk of NMOSD. We found that MHC class I-related biological processes may be involved in the pathogenesis of NMOSD coexisting with other ADs. We also identified 30 chemicals that might have therapeutic effects. However, our study has several limitations. First, the sample size of the NMOSD trait is relatively small, so we adopted a relaxing p value of 1E-6 to screen for more exposure IVs, but this would compromise the reliability of our MR results. However, we mainly focused on the exposure of AITD, SLE, and SS, which have robust sample sizes for analysis. Second, performing drug prediction by only using causal SNPs would leave out some important therapies, such as IL-6R mAbs and complement inhibitors. Third, our results lack validation in the clinical cohort and basic research. Since bioinformatic studies only provide predictions; our results should be interpreted with caution.

In the present study, we provide evidence from genetic levels, but we lack experimental validation. Future studies need to address several questions. First of all, it is unknown whether a common trigger, such as a viral infection, is involved in the pathogenesis of NMOSD and other ADs. Secondly, there is a need to clarify what causes the disruption of the blood–brain barrier during the pathogenesis of systemic autoimmune diseases, since myelitis has also been observed in SLE despite the presence of NMOSDs. Thirdly, it is urgent to determine the best treatment strategy for NMOSD patients coexisting with other ADs, since some biotherapy (e.g., anti-TNFa) may lead to demyelination and increase the disability of NMOSD. Furthermore, prospective cohorts with large sample sizes may be needed to evaluate the sequence of onset of NMOSD and other ADs.

## Conclusion

By using GWAS summary data and MR analysis, we identified causal associations of AITD, SLE, and SS with increased risk of NMOSD. We found that MHC class I-related biological processes and interferon-gamma-mediated signaling pathway may be involved in the pathogenesis of NMOSD coexisting with AITD, SLE, and SS. These findings could help better understand the etiology of NMOSD and provide potential therapeutic targets for patients with coexisting conditions.

## Data availability statement

The original contributions presented in the study are included in the article/**Supplementary Material**. Further inquiries can be directed to the corresponding author.

## Author contributions

XW wrote the main manuscript text and prepared the tables. ZS and ZZ prepared figures. HC, YL, and LK managed the data. XL, QD, and JW revised the manuscript. HZ provided the conception of this study and supervised this study. All authors reviewed the manuscript. All authors contributed to the article and approved the submitted version.

## Funding

This work was funded by the Natural Science Foundation of Sichuan Province (Grant No. 2022NSFSC1432 to Xiaofei Wang and 2022NSFSC1591 to Zhengyang Zhao), Department of Science and Technology of Sichuan Province (Grant No. 2020YFS0219 to Ziyan Shi and 2021YFS0173 to Hongyu Zhou), and the 1·3·5 project for disciplines of excellence–Clinical Research Incubation Project, West China Hospital, Sichuan University (Grant No. 21HXFH041 to Hongyu Zhou).

## Acknowledgments

We thank Figdraw (www.figdraw.com) for plotting **Figure 6**.

## Conflict of interest

The authors declare that the research was conducted in the absence of any commercial or financial relationships that could be construed as a potential conflict of interest.

## Publisher’s note

All claims expressed in this article are solely those of the authors and do not necessarily represent those of their affiliated organizations, or those of the publisher, the editors and the reviewers. Any product that may be evaluated in this article, or claim that may be made by its manufacturer, is not guaranteed or endorsed by the publisher.

## Supplementary material

Additional file 1 F-statics, annotation, and cis-eQTL results of the causal SNPs.

Additional file 2 GO and KEGG pathway enrichment results.

Additional file 3 Prediction of therapeutic chemicals using Comparative Toxicogenomics Database.

## Abbreviations

AD, autoimmune Disease; AITD, autoimmune thyroid disease; cis-eQTL, cis-expression quantitative trait loci; CI, confidence interval; DMF, dimethyl fumarate; GO, Gene Ontology; GWAS, genome-wide association; IV, instrumental variable; IVW, inverse variance weighted; MHC, major histocompatibility complex; MR, Mendelian randomization; MS, multiple sclerosis; NMOSD, neuromyelitis optica spectrum disorder; SLE, systemic lupus erythematosus; SS, Sjogren’s syndrome; SNP, single-nucleotide polymorphism.
